# Antifungal Plant Defensins: Mechanisms of Action and Production

**DOI:** 10.3390/molecules190812280

**Published:** 2014-08-14

**Authors:** Kim Vriens, Bruno P. A. Cammue, Karin Thevissen

**Affiliations:** 1 Centre of Microbial and Plant Genetics, Katholieke Universiteit Leuven, Kasteelpark Arenberg 20, Heverlee 3001, Belgium; 2 Department of Plant Systems Biology, VIB, Technologiepark 927, Ghent 9052, Belgium

**Keywords:** mechanism of action, antimicrobial peptide, plant defensin, RsAFP2, NaD1, MsDef1, MtDef4, Psd1, heterologous protein expression, chemical protein synthesis

## Abstract

Plant defensins are small, cysteine-rich peptides that possess biological activity towards a broad range of organisms. Their activity is primarily directed against fungi, but bactericidal and insecticidal actions have also been reported. The mode of action of various antifungal plant defensins has been studied extensively during the last decades and several of their fungal targets have been identified to date. This review summarizes the mechanism of action of well-characterized antifungal plant defensins, including RsAFP2, MsDef1, MtDef4, NaD1 and Psd1, and points out the variety by which antifungal plant defensins affect microbial cell viability. Furthermore, this review summarizes production routes for plant defensins, either via heterologous expression or chemical synthesis. As plant defensins are generally considered non-toxic for plant and mammalian cells, they are regarded as attractive candidates for further development into novel antimicrobial agents.

## 1. Introduction

Like all living organisms, plants are repeatedly confronted with attacks by for instance insects, fungi and bacteria. In order to cope with these pests and pathogens, plants have developed a number of defence mechanisms, including the production of antimicrobial peptides (AMPs). Expression of these AMPs can be constitutive in e.g., storage organs and reproductive tissues or can be induced systemically as well as locally, in e.g., leaves, during microbial invasion or injury [1,2,3]. Plant AMPs are small cationic peptides that exert biological activity against a broad range of organisms. Their activity is primarily directed against fungi, but bactericidal and insecticidal actions are also reported. These defence-related peptides have a compact structure that is stabilized by intramolecular disulphide bridges, enhancing structural and thermodynamic stability [2]. Based on their tertiary structure, they are subdivided into distinct classes, being thionins, defensins, knottins, lipid transfer proteins, heveins, snakins and cyclotides (reviewed in [2,4,5,6,7]). As the scope of this review is focused on plant defensins, the other classes of AMPs will not be discussed. For more detailed information on antifungal AMPs, the reader is referred to [8,9,10].

## 2. Plant Defensins

Plant defensins are present in all plant families, including the *Brassicaceae,* *Fabaceae* and *Solanaceae*. These peptides were primarily found in the seeds, but leaves and flowers are also common sources [11,12,13,14,15,16]. They are either constitutively expressed in storage and reproductive organs or produced upon pathogenic attack or injury as part of a systemic defence response [2]. In addition, production of plant defensins is also induced in response to environmental stress, such as drought [17,18], and signalling molecules, including methyl jasmonate, ethylene and salicylic acid [19,20].

### 2.1. Structure

Plant defensins are small, cationic peptides with a length of approximately 45–54 amino acids. Their structure typically comprises a cysteine-stabilized αβ-motif (CSαβ) with a prominent α-helix and a triple-stranded antiparallel β-sheet that is stabilized by four disulphide bridges [21,22,23]. A subclass of the plant defensin family comprises defensins with 10 cysteine residues, resulting in a total of five disulphide bonds. The fifth disulphide bond seems to reinforce a conserved hydrogen bond and is likely to confer additional thermodynamic stability of the defensin, as compared to other defensins, by replacing non-covalent hydrophobic interactions or hydrogen bonds with a covalent bond [24]. To our knowledge, this extra pair of cysteines has only been reported for PhD1 and PhD2, both floral defensins isolated from *Petunia* *hybrida* [16,24].

According to the structure of their precursor protein, plant defensins can be subdivided into two groups. A first group comprises defensins in which the precursor is composed of a signal sequence and a mature defensin domain. The signal sequence targets the protein to the endoplasmic reticulum, where it is folded and subsequently enters the secretory pathway. In a second and less common group, the precursor protein contains an additional C-terminal prodomain that is proteolytically removed during or after transit through the secretory pathway [16,25]. This type of defensins have been identified in solanaceous plants, such as *Nicotiana alata* and *Petunia hybrida* [16]. Recently, Lay and co-workers assigned a cytoprotective and subcellular targeting function to this prodomain [26].

### 2.2. Biological Activity

Plant defensins possess a variety of biological activities (reviewed in [27,28,29]). They have been reported to inhibit protein synthesis, enzyme activity and ion channel function. Some plant defensins even exhibit antiproliferative activity towards cancer cells or proved effective against HIV reverse transcriptase. To date, only a few plant defensins are shown to inhibit the growth of bacteria, whereas their antifungal activity has been studied extensively [27,28]. It has become clear that, although a similar activity might be observed for several defensins, their mode of action can be extremely diverse with regard to target molecules and (sub)cellular localization [30]. In order to illustrate this variety, this review will focus on the mode of antifungal action of plant defensins isolated from *Raphanus* *sativus* (RsAFP2), *Pisum* *sativum* (Psd1), *Medicago* spp. (MsDef1 and MtDef4) and *Nicotiana* *alata* (NaD1).

## 3. Mode of Action of Plant Defensins

Extensive research during the past decades has allowed us to identify a number of key features by which plant defensins exert their antimicrobial activity. It has been demonstrated that defensins can specifically interact with host membrane compounds, such as bacterial lipid II receptors (reviewed in [31]), fungal sphingolipids (reviewed in [31]) and fungal phospholipids [32]. Fungal sphingolipids are classified into two groups, *i.e.*, phosphosphingolipids and glycosphingolipids (GSLs). The most common GSL is glucosylceramide (GlcCer), which is synthesized by glucosylceramide synthase, encoded by the *GCS* gene, and glycoinositolphosphorylceramide (GIPC) [33,34]. The latter can be further subdivided into inositolphosphorylceramide (IPC), mannosyl-IPC (MIPC) and mannosyldi-IPC (M(IP)_2_C), which are formed by sequential addition of inositolphosphate and mannose. The final step in this reaction, *i.e.*, converting MIPC to M(IP)_2_C, requires inositolphosphate transferase, encoded by the *IPT1* gene (reviewed in [35]). Different plant defensins have been shown to interact with different classes of sphingolipids: the plant defensin RsAFP2 from radish [11] interacts with GlcCer [36], whereas the plant defensin DmAMP1 from dahlia [37] interacts with M(IP)_2_C [38,39]. In contrast, the plant defensins NaD1 from tobacco was recently shown to interact with a variety of phospholipids, including phosphatidylinositol mono-/bis-/tri-phosphates, phosphatidylserine and phospatidic acid, but not with sphingolipids [32]. In case of plant defensins that interact with sphingolipids, it was found that the presence of specific sphingolipids was essential mediating cell death of fungi and yeast, since yeast mutants deficient in genes involved in sphingolipid biosynthesis were resistant towards these peptides. For instance, *S. cerevisiae* strains with a non-functional *IPT1* allele, and thus lacking M(IP)_2_C in their membranes, were found highly resistant towards DmAMP1 as compared to the wild type (WT) [38]. In line, deletion of the *GSC* gene resulted in an increased resistance towards RsAFP2 for *Pichia pastoris* and *Candida albicans* knockout strains as compared to the corresponding WTs [36]. Similar observations were made for MsDef1 and a Δgcs *Fusarium graminearum* strain [40]. Furthermore, *Neurospora crassa* mutants displaying structurally different GlcCer, novel glycosphingolipids and an altered level of steryl glucosides in their membranes were found more resistant towards RsAFP2, DmAMP1 and HsAFP1 when compared to the WT, suggesting that the specific structure of sphingolipids in the fungal membrane is crucial for sensitivity towards plant defensins [41]. In addition, interaction of Sd5 and Psd1 with respectively fungal GlcCer- [42] and phosphatidylcholine- (PC) [43] containing vesicles further highlights the importance of fungal sphingolipids as interaction sites for plant defensins.

Upon interaction with their target, plant defensins are either internalized by the fungal cell and interact with intracellular targets, or they stay at the cell surface and induce cell death through induction of a signalling cascade. While the latter has been reported for RsAFP2 [44], cellular uptake was observed for NaD1 [45,46], MtDef4 [47] and Psd1 [48]. Recently, Sagaram and colleagues identified a RGFRRR motif in the MtDef4 sequence that is thought to be a translocation signal required for fungal cell entry, since replacement of this motif with AAAARR or RGFRAA abolished the ability of the peptide to enter the cell [47]. However, this sequence is, to our knowledge, not present in other plant defensins that enter the fungal cell. This suggests multiple mechanisms by which defensins are internalized by the cell. Proposed mechanisms include receptor-mediated internalization, membrane translocation (*i.e.*, transient membrane permeabilization and lipid-assisted pore formation) and membrane permeabilization (reviewed in [49]). Membrane permeabilization has been described for plant defensins, however, it is suggested to be a secondary effect rather than the key to microbial killing, since it only occurs at concentrations well above the concentration required for growth inhibition [50]. Alternatively, ROS production and hence oxidative stress, most often play a role in defensin-mediated cell death, as has been reported for many plant defensins including RsAFP2 [51], HsAFP1 [52], DmAMP1 [53] and NaD1 [45].

Another common feature of plant defensins is the loss of their antifungal activity by an increase in ionic strength of the growth medium. Especially divalent cations, such as Ca^2+^ and Mg^2+^, seem to play an important role in this phenomenon [11,13,15,37,54]. Since the antagonistic effect strongly depends on the test fungus and type of the defensin, it is suggested that electrostatic interactions alter the target site on the fungal membrane, and hence reduce the affinity of the defensin to bind the membrane, rather than altering the conformation of the defensin itself [55]. However, Oard and Karki proposed another mechanism for inhibition of antimicrobial activity by cations which is in contrast with this interpretation. They found that the structure of β-purothionin, a thionin purified from wheat, is altered by the presence of K^+^ and Mg^2+^, making the peptide more rigid and impairing interaction with its target. Structural changes in this case include elongation of α1-helix, unfolding of α2-helix and an overall change in loop conformation [56]. Inhibition of antimicrobial activity by the presence of cations seems to be a common theme for AMPs in general, since it is also observed for thionins, insect defensins and mammalian defensins and is not solely associated with antifungal activity [56,57,58,59,60]. 

**Figure 1:**
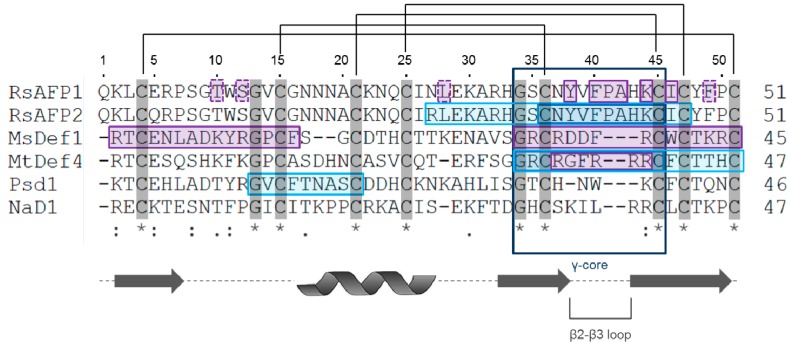
Amino acid sequence alignment of RsAFP1, RsAFP2, MsDef1, MtDef4, Psd1 and NaD1. Multiple alignment was performed using the alignment tool from UniProt. Cysteine-pairing is shown at the top of the figure. Highly conserved residues are shown in grey; (-) denote gaps in the alignment. Arrows represent the position of the β-strands; the helix represents the position of the α-helix. Purple boxes indicate regions important for antifungal activity: boxes with dashed and full lines in the RsAFP1 sequence represent the first and second site in the tertiary structure, resp., important for antifungal activity [15,47,63]; blue boxes represent peptide fragments that exhibit antifungal activity similar to the parental peptide and hence, are important for antifungal activity [43,47,63,64].

In order to illustrate the variety of mechanisms of action by which plant defensins exhibit their antifungal activity, four case studies will be discussed in the next sections. The plant defensins discussed include RsAFP1 and RsAFP2 from radish [11], Psd1 from pea pods [13], MsDef1 from alfalfa [14] and MtDef4 from barrel clover [61], and NaD1 from tobacco [16] and are listed in Table 1. A multiple alignment of the amino acid sequences of these defensins is presented in Figure 1, in which the regions important for their antifungal activity are highlighted.

**Table 1:** Overview of the plant defensins discussed in this review. NA: not available.

Defensin Name	Source	UNIPROT Accession Number	Protein Data Bank Accession Number	Reference
RsAFP1	Radish seeds	P69241	1AYJ	[11]
RsAFP2	Radish seeds	P30230	NA	[11]
MsDef1	Alfalfa seeds	Q9FPM3	1H3R (theoretical model)	[14]
MtDef4	Barrel clover seeds	G7L736	2LR3	[61]
Psd1	Pea pods	P81929	1JKZ	[13]
NaD1	Tobacco flowers	Q8GTM0	1MR4	[62]

**Figure 2:**
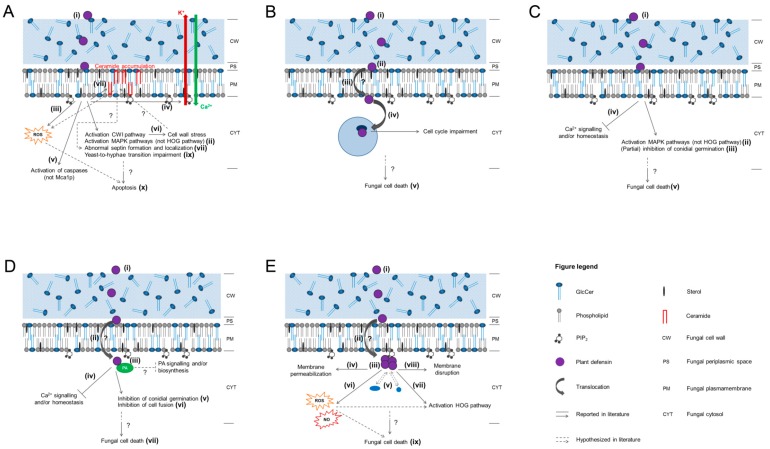
Schematic overview of the proposed mechanisms of action of the plant defensins discussed in this review. (**A**) RsAFP1 and RsAFP2; (**B**) Psd1; (**C**) MsDef1; (**D**) MtDef4; (**E**) NaD1. For detailed information on the Roman numerals displayed in the figures, the reader is referred to Section 3.1 (**A**), 3.2 (**B**), 3.3 (**C**), 3.4 (**D**) and 3.5 (**E**) of this review.

A schematic overview of the proposed mechanism of action of the plant defensins discussed in this review is given in Figure 2.

### 3.1. Plant Defensins from Radish: RsAFP1 and RsAFP2

RsAFP1 and RsAFP2 are antifungal defensins found in the seeds of radish [11,36,65]. Regarding their antifungal activity, it was shown that RsAFP2 is more potent than RsAFP1 (2-30-fold dependent on the test fungus), although differences between the two peptides solely consist of two amino acid substitutions [11]. Analysis of the RsAFP2 primary structure showed two adjacent sites involved in antifungal action.

These two regions might constitute two sites contacting a single receptor/interaction site or might indicate two binding sites that interact with two receptor/interaction sites [66]. The membrane target of RsAFP2 has been identified as fungal GlcCer [36] and since sphingolipids, such as GlcCers, are clustered with other membrane compounds to form lipid rafts [67], the hypothesis of RsAFP2-interaction with multiple interaction sites is plausible.

Interaction of RsAFP2 with its membrane target is essential, however not sufficient for antifungal activity, as [Y38G]RsAFP2, an RsAFP2 variant devoid of antifungal activity, is able to interact with GlcCer [36,66]. Other RsAFP2-associated aspects leading to fungal cell death have been described. Ion fluxes take part in RsAFP2-induced cell death, since a rapid K^+^ efflux and Ca^2+^ influx was observed in RsAFP2-treated *N.* *crassa* hyphae [50]. Moreover, Ca^2+^ influx has been correlated with the antifungal potency of RsAFP2 [66], however, no blockage of L-type Ca^2+^ channels is observed [15]. In addition, production of ROS was shown in RsAFP2-treated *C. albicans* cells, suggesting a downstream signalling cascade of RsAFP2-binding to GlcCer [51]. In line with these findings, Aerts and colleagues demonstrated that RsAFP2 induces programmed cell death or apoptosis in *C. albicans*. Moreover, RsAFP2-induced apoptosis involves caspases, but not metacaspase Mca1 [68].

Cell wall stress has been associated with RsAFP2 activity as well. As GlcCers are also abundant in the cell wall (*i.e.*, approx. 40% of GlcCer is located in the cell wall) [44], this is not surprising. RsAFP2 activates the cell wall integrity pathway by increasing the level of dually phosphorylated Mkc1p [44], a MAP kinase in *C. albicans* associated with oxidative stress, changes in osmotic pressure, cell wall damage and a decrease in growth temperature [69]. In line, RsAFP2 was shown to activate MAP kinase signalling cascades in *F. graminearum*, without involvement of the Hog1 MAP kinase pathway [61]. Furthermore, RsAFP2 was found to induce accumulation of membrane phytoC24-ceramides, affect septin formation and localization and impair the yeast-to-hyphae transition in *C. albicans* [44]. 

The exact binding site of RsAFP2 to GlcCer is still unknown, however, the region encompassing the β2-β3 loop is suggested to play a role in binding to the fungal membrane, as synthetic derivatives of this region, displayed in Figure 1, exhibited antifungal activity and binding to the membrane is essential to induce fungal cell death [64,66,70]. Furthermore, position 38 and 39 were shown critical for antifungal action, as amino acid substitutions at these positions significantly altered the potency of the peptide *in vitro* [66]. Interestingly, [Y38G]RsAFP2, which is devoid of antifungal activity, is still able to interact with its membrane target, and hence, target binding and antifungal activity seem not controlled by the same peptide region [36]. Noteworthy is the fact that loop regions have been demonstrated to be important for antifungal activity in other plant defensins as well, including Psd1, MsDef1 and MtDef4 [15,43,63].

In conclusion, the mode of action of RsAFP2 is suggested to involve (i) recognition of and binding with GlcCer in the fungal membrane and cell wall; (ii) activation of the CWI pathway and MAP kinase signalling pathways, excluding the Hog1 MAP kinase pathway; (iii) production of ROS; (iv) induction of ion fluxes; (v) activation of caspases, but not metacaspase; (vi) induction of cell wall stress; (vii) accumulation of membrane phytoC24-ceramides; (viii) abnormal septin formation and localization; (ix) impairment of the yeast-to-hyphae transition and (x) fungal cell death. In which order step (ii) to (ix) take place, has yet to be elucidated. A schematic overview of the proposed mechanism of action of RsAFP2 can be found in Figure 2A.

### 3.2. Plant Defensin from Pea: Psd1

Psd1 is an antifungal defensin isolated from pea seeds [13]. Almeida and colleagues estimated other biological activities of Psd1 based on *in silico* analysis of the surface topology using various well-characterized defensins and toxins. Although no correlation could be demonstrated between antifungal or antibacterial activity and the surface electrostatic potential of the considered peptides, the study showed a clear correlation between K^+^ channel inhibitory activity and peptide surface topology. Since Psd1 shows a surface topology similar to that of peptides and toxins belonging to the latter group, it is therefore implied to act as a K^+^ channel inhibitor [71]. 

The fungal membrane target of Psd1 has not yet been identified, but is suggested to be GlcCer and/or ergosterol [43,72]. Analysis of the Psd1 lipid selectivity by fluorescence spectroscopy revealed that the peptide is likely to be adsorbed on or slightly inserted into the fungal membrane during initial interaction. Furthermore, this study showed that Psd1 does not interact with membranes containing GlcCer derived from soybean, nor with cholesterol-enriched lipid bilayers, such as mammalian cell membranes, which highlights its therapeutic potential [72]. The exact binding site for membrane interaction is thought to be Psd1 Loop1, displayed in Figure 1, since the peptide corresponding to this region still interacts with GlcCer-containing vesicles. Moreover, this region was found to exhibit significant conformational changes upon binding with GlcCer, as compared to the conformational accommodation during nonspecific binding to phosphocholine. Hence, interaction of Psd1 with the fungal membrane is probably due to conformational selection [43].

Upon membrane binding, Psd1 is internalized by the cell and interacts with nuclear proteins, as was shown via a GAL4-based yeast two-hybrid assay by Lobo and colleagues [48]. Cyclin F was identified as the main target of Psd1, which plays a key role in nuclear translocation of Cyclin B and cell cycle progression [48,73,74,75]. *In vivo* studies with Psd1 on retinal neuroblasts showed that Psd1 affects interkinetic nuclear migration and hence, impairs cell cycle progression [48].

Taken together, the mechanism of action of Psd1 is not yet completely understood, however, a proposed mode of action includes (i) adsorption on the fungal membrane surface; (ii) interaction with GlcCer and/or ergosterol in the fungal membrane; (iii) insertion of Psd1 into the fungal membrane; (iv) nuclear translocation and interaction with Cyclin F, resulting in cell cycle impairment and (v) fungal cell death. In which order step (iii) to (v) take place has yet to be identified. A schematic overview of the proposed mechanism of action of Psd1 is represented in Figure 2B.

### 3.3. Plant Defensins from Medicago spp.: MsDef1 and MtDef4

MsDef1 and MtDef4 are antifungal defensins found in *Medicago* spp. [14,61]. MsDef1 is suggested to display a similar mode of antifungal action as the virally encoded toxin KP4 from the fungus *Ustilago* *maydis*, since both peptides strongly block the mammalian L-type Ca^2+^ channel in a specific manner [15]. Interaction of toxins with K^+^ channels has been reported by Zhu and colleagues, who hypothesized that defensins with a Lys-Cys4-Xaa-Asn motif interact with K^+^ channels when the flexible loop of the peptide is deleted and hence, steric hindrance is reduced [76]. The same might hold true for other ion channels, such as Ca^2+^ channels. Similarities in modes of action between KP4 and MsDef1 are further demonstrated by a more pronounced hyperbranching of fungal hyphae upon treatment with these peptides as compared to treatment with RsAFP2 [15]. Moreover, the antifungal activity of MsDef1 and KP4 is strongly abrogated by addition of exogenous Ca^2+^ and since addition of other metals (K^+^, Mg^2+^ and Na^+^) did not affect peptide activity, Ca^2+^ is suggested to be involved in MsDef1 mode of action [15]. Recently, Muñoz and colleagues confirmed that MsDef1, as well as MtDef4, disrupt Ca^2+^ signalling and/or homeostasis and that this phenomenon is not caused by direct membrane permeabilization [77].

*GCS* was found essential in MsDef1-mediated growth inhibition, which implies the involvement of GlcCer as a fungal membrane target [40]. This is in line with recent findings by Muñoz and colleagues who concluded that MsDef1-sensitivity is mediated by GlcCer in filamentous fungi [77]. Molecular targets involved in MsDef1-tolerance include MAP kinase signalling cascades, with exception of the Hog1 MAP kinase pathway, and were similar to the observations made for RsAFP2. Immunoblot analysis revealed rapid activation of Gpmk1 and Mgv1 MAP kinases upon MsDef1 treatment [61], which regulate processes related to cell wall integrity, sexual reproduction and pathogenicity [78,79]. Interestingly, RsAFP2 activates the cell wall integrity pathway and exerts its antifungal activity from the extracellular space [44]. Since MsDef1 and RsAFP2 seem to activate the same MAP kinase signalling cascades [61], they are suggested to have a similar mode of action. Hence, MsDef1 is suggested to induce fungal cell death through activation of signalling cascades involving MAP kinases without entering the fungal cell.

In contrast to MsDef1 activity, *GCS* is not important for MtDef4-mediated fungal growth inhibition and MtDef4 activity seems independent of MAP kinase signalling cascades. Furthermore, MsDef1 induces extensive hyperbranching of fungal hyphae, whereas MtDef4 does not, suggesting different mechanisms of action for MsDef1 and MtDef4 [61]. Both peptides were shown to induce membrane permeabilization, however, membrane permeabilization was significantly higher in hyphae treated with MtDef4 as compared to treatment with MsDef1, which is consistent with the *in vitro* antifungal potency of the peptides [63]. Sagaram and colleagues reported internalization of MtDef4 in the fungal cell and identified the RGFRRR motif in the MtDef4 sequence as a translocation signal that is required for fungal cell entry. In addition, MtDef4 was shown to interact with cytosolic phosphatidic acid (PA), dependent on the presence of the RGFRRR motif [47]. These results are consistent with previous findings that correlate the antifungal activity of MtDef4 with the presence of that motif [63]. Recently, Muñoz and colleagues reported that MsDef1 and MtDef4 affect conidial germination, conidial anastomosis tube fusion and conidial cell death in *N. crassa* in significantly different ways. High fungicidal concentrations of MtDef4 caused rapid and complete inhibition of germination, with cell death occurring rather fast, whereas treatment of conidia with high MsDef1 concentrations resulted in a delayed cell death and complete inhibition of germination was not reached. Moreover, it was shown that the RGFRRR motif in MtDef4 is not only important for cell entry and binding to PA, but it furthermore plays a role in inhibition of cell fusion [77].

As is the case for various plant defensins, structure-activity studies have been performed for MsDef1 and MtDef4. Both the N-terminal and the C-terminal region of MsDef1, displayed in Figure 1, were found important for antifungal activity and, in line with the observations made for RsAFP2, position 38 is critical for antifungal action [15,66]. The MsDef1 and MtDef4 C-terminal domains are also suggested to play a role in Ca^2+^ homeostasis [77]. Furthermore, Spelbrink and colleagues demonstrated the importance of the loops for antifungal activity in the MsDef1 tertiary structure, which has been reported for Psd1, RsAFP2 and MtDef4 as well [15,43,63,66,70]. Structure-activity determinants of MtDef4 demonstrated the importance of the γ-core motif, composed of β2 and β3 strands and the interposed loop, for antifungal activity. Here, cationic and hydrophobic residues are considered important for antifungal action, as the MtDef4 γ-core alone is able to inhibit fungal growth, whereas the γ-core of MsDef1 is not. Both F_37_ and R_38_ are critical for antifungal activity in MtDef4, since the hexapeptide RGFRRR present in the MtDef4 γ-core is capable of inducing growth inhibition and membrane permeabilization, while RGARRR and RGFARR are not [63]. In addition, replacement of RGFRRR with RGFRAA or AAAARR abolished the ability of the peptide to enter the fungal cell as well as to interact with intracellular PA, suggesting that both fungal cell entry and PA binding are mediated by the RGFRRR loop [47], again highlighting the importance of peptide loops for antifungal activity.

In conclusion, MsDef1 and MtDef4 seem to have different mechanisms of antifungal action, however, their complete mode of action remains to be elucidated. The proposed mechanism of antifungal action for MsDef1 includes: (i) interaction with the fungal membrane, presumably GlcCer; (ii) activation of Gpmk1 and Mgv1 MAP kinase signalling cascades; (iii) (partial) inhibition of conidial germination; (iv) disruption of Ca^2+^ signalling and/or homeostasis and (v) delayed fungal cell death. In which order step (ii) to (v) take place has yet to be identified. The hypothesized mechanism of action of MtDef4 comprises (i) recognition of the fungal membrane; (ii) translocation of the peptide to the cytosol via the RGFRRR motif; (iii) interaction with cytosolic PA and supposedly subsequent interference with PA signalling and/or biosynthesis; (iv) disruption of Ca^2+^ signalling and/or homeostasis; (v) inhibition of conidial germination; (vi) inhibition of cell fusion and (vii) rapid fungal cell death. In which order (ii) to (vii) take place remains to be elucidated. A schematic overview of the proposed mechanism of action of MsDef1 and MtDef4 is given in Figure 2C,D, respectively.

### 3.4. Plant Defensin from Tobacco: NaD1

NaD1 is a floral defensin from the tobacco plant and exhibits antifungal properties [16]. The membrane target of NaD1 was recently identified by Poon and colleagues as the phospholipid PIP_2_, which is present in eukaryotic cell membranes. It was shown that NaD1 forms 14-mer oligomers, mediated by PIP_2_, and that this oligomerization is important for membrane permeabilization and lysis of the fungal cell [32]. Upon interaction and permeabilization of the cell membrane, NaD1 enters fungal hyphae and is localized to the cytoplasm, where it causes granulation of the cytoplasm and induces ROS production [45]. NaD1 is implied to induce cell death via oxidative stress, as ROS and nitric oxide (NO) production was observed upon treatment of *C. albicans* cells [80]. These findings are consistent with results reported by Bleackley and colleagues, in which mitochondrial genes are implicated in NaD1 mode of action [81].

Recently, Hayes and colleagues reported the importance of the high-osmolarity glycerol (HOG) pathway in tolerance to NaD1, being the sole stress-responsive pathway involved in NaD1 action that was screened in this study [80]. Although the HOG pathway is mainly involved in protection against osmotic stress and osmotic stress does not contribute significantly to NaD1 mode of action [80], these findings are not surprising, since Hog1p is also known to participate in tolerance to oxidative stress [82]. Interestingly, the HOG pathway is excluded in the mode of action of MsDef1 and RsAFP2, whereas other MAP kinase signalling cascades play a key role in tolerance to these defensins [61]. These findings clearly suggest distinct modes of action of MsDef1 and RsAFP2 on the one hand, and NaD1 on the other. Another novel finding in the NaD1 mechanism of action is the identification of Agp2p as a regulator of the potency of the peptide [81]. Agp2p is a plasma membrane protein that regulates the transport of positively charged molecules. Upon NaD1 treatment, cells lacking *AGP2* show a delayed membrane permeabilization, reduced uptake of NaD1 and are overall more resistant to NaD1 treatment compared to the WT. Deletion of *AGP2* probably results in an accumulation of positive charges on the surface of the cell, thereby repelling cationic peptides from the surface [81].

Taken together, the NaD1 mechanism of action includes (i) interaction with the fungal cell membrane; (ii) translocation to the cytoplasm; (iii) PIP_2_-mediated oligomerization of NaD1 (14-mer) (iv) membrane permeabilization; (v) possible interaction with intracellular targets; (vi) ROS and NO production, *i.e.*, oxidative stress; (vii) activation of the HOG pathway; (viii) membrane disruption and (ix) fungal cell death. In which order (ii) to (ix) take place has yet to be identified. A schematic overview of the proposed mechanism of action of NaD1 is shown in Figure 2E.

## 4. Production of Plant Defensins

Due to their selective toxicity towards microbial cells and their unique mode of action, plant defensins are attractive candidates for further development as novel antimicrobial agents. However, development of plant defensins for medicinal or biotech purposes requires large amounts of purified peptides. Extraction of plant defensins from natural sources is rather complicated due to their low abundance and the presence of a variety of other compounds in these plant parts. Chemical synthesis and heterologous production are therefore convenient alternatives to obtain large amounts of functional peptides. In addition, these approaches allow for the production of mutant peptides, which are interesting to include in structure-activity studies. In the following part, these techniques as well as their advantages and drawbacks are discussed.

### 4.1. Chemical Synthesis of Proteins

Synthesis of proteins by chemical means has recently gained interest, as it allows the generation of proteins that cannot be produced biologically, e.g., labelled peptides. Various strategies have been developed in which proteins can be synthesized, often consisting of combinations of solid-phase peptide synthesis (SPPS), native chemical ligation (NCL) and enzyme-catalyzed ligation (reviewed in [83,84,85,86]).

In SPPS, a peptide is synthesized in a stepwise manner on a polymeric resin through sequential steps of coupling and deprotection of protected amino acids. Both Fmoc (9-fluorenylmethyl carbamate) and Boc (di-tert-butyl dicarbonate) strategies can be used in SPPS to protect the N-terminus of the amino acid being coupled and hence prevent polymerization or non-specific reactions (reviewed in [87,88,89]. SPPS plays a key role in peptide synthesis, however, studies have shown that following this method, only peptides containing less than 50 amino acids can be reliably prepared with acceptable yields and purity [83]. Hence, other strategies have to be implemented when synthesizing larger peptides and proteins. To this end, a strategy of peptide segment condensation is used in which unprotected peptide fragments, often produced by SPPS, are subjected to ligation methods such as NCL. Subsequent cycles of NCL results in the formation of a linear polypeptide chain [83,90,91]. A major drawback of this technique is the mandatory use of an N-terminal cysteine residue, as cysteines are seldom conveniently distributed throughout a peptide sequence. Furthermore, the polypeptide is generated from the C-terminus towards the N-terminus and not *vice versa* [85]. Another approach to couple two peptides includes enzyme-assisted ligation, in which enzymes are used as catalysts to promote peptide bond formation. This approach complements chemical ligation strategies and has great significance since these enzymes are naturally involved in protein modifications *in vivo* and are rated nontoxic, whereas many chemicals employed in NCL are unfavourable for food applications of the peptides. Nonetheless, the use of enzymes in protein crosslinking is still in its infancy and further research is essential to improve enzyme-assisted peptide ligation (extensively reviewed in [84]). 

Proteins often require post-translational and conformational modifications in order to render biological activity. As post-translational modifications cannot be provided via chemical synthesis, a strategy termed expressed protein ligation (EPL) or intein-mediated protein ligation (IPL) is employed. Following this method, semisynthetic peptides are created by fusion of recombinant peptide fragments to synthetic peptide fragments. Since post-translational modifications mainly occur at the termini of peptides, EPL is a plausible approach to obtain functional peptides [91,92]. When a disulphide bond pattern is essential, (re-)folding of the protein is advised and oxidative folding is performed [93,94,95,96]. In a first attempt, oxidative folding is often employed in a direct manner, *i.e.*, a one-step oxidative folding procedure, as it allows for a spontaneous fold in which the protein is energy-stable and assumed to acquire its native conformation. In addition, it is less expensive and time-consuming as compared to regioselective oxidative folding in which individual cysteine pairs are deprotected and oxidized sequentially to allow subsequent formation of disulphide bonds [97].

Functional peptides can be synthesized following SPPS and NCL methods, as was reported for conotoxins, snakins and defensins [94,95,97,98,99]. For instance, the insect defensin lucifensin, synthesized by Fmoc-SPPS and folded using a one-step oxidative folding technique, showed biological activity against *Bacillus subtilis*, *Micrococcus luteus* and *Staphylococcus aureus* with MIC-values of 1.2 µM, 0.6 µM and 41 µM, resp., whereas linear unfolded lucifensin and lucifensin analogues folded by 1 out of 3 disulphide bridges were inactive (MIC > 100 µM) [99]. Furthermore, human β-defensin 4 (HBD4) and HBD4 analogues, synthesized using Fmoc-SPPS and folded employing a three-step oxidative folding procedure, showed antimicrobial activity against *Escherichia coli*, *Pseudomonas aerigunosa*, *S. aureus* and *C. albicans*. However, only the completely folded peptide showed a similar or a 2-fold decreased activity, depending on the test organism, as commercially available HBD4 [98]. Although it seems that plant defensins can be produced by chemical means, multidisulphide-containing peptides are not always successfully produced, as multiple isoforms are generated during folding [96]. In addition, chemical synthesis is rather expensive due to a high cost of reagents, and peptide aggregation and formation of by-products renders this method often unfavourable [100,101]. These observations highlight the need for other systems to generate functional proteins, as will be discussed in the next section.

### 4.2. Heterologous Expression of Proteins

Heterologous expression of proteins is a widely used technique and different expression systems have been reported to date. The main host systems used for recombinant production of AMPs include *Escherichia coli* and *Pichia* *pastoris* (reviewed in [102]). 

#### 4.2.1. Heterologous Expression of Proteins in *E. coli*

Protein expression in *E. coli* is relatively simple and inexpensive and the variety of available plasmids, fusion partners and strains makes it often the preferred method for production of AMPs [103,104]. However, major drawbacks have been reported using bacteria for effective AMP production as discussed below.

First of all, the recombinant protein often needs to be fused to a carrier protein to neutralize its toxicity towards the host and to increase its solubility to avoid formation of inclusion bodies [105]. This fusion partner needs to be released during or after purification of the protein of interest via enzymatic or chemical cleavage to render functional proteins, which results in a decreased yield [102]. In addition, fusion proteins are not necessarily properly folded and production of these proteins can result in so-called “soluble inclusion bodies” [105]. Recently, it was shown that co-expression of the human Quiescin Sulfhydryl Oxidase (QSOX), a chaperone with thiol/disulphide oxidase activity, in the cytoplasm of *E. coli* can counter protein misfolding and increase the yield of soluble cysteine-rich proteins [106,107]. Although such approach improves protein production in *E. coli*, other obstacles remain.

Direct secretion mechanisms are not present in *E. coli* strains used for recombinant protein production, which complicates protein purification. Protein secretion can be obtained, however, by destabilization of the *E. coli* structural components or by using leaky strains that lack certain structural components or mutant strains in which secretion modules derived from pathogenic *E. coli* or other species are incorporated [103,108,109,110,111].

Finally, and most importantly, the protein of interest often requires complex folding, including the formation of multiple disulphide bonds and/or glycosylation. In both cases, an eukaryotic system is preferred [112]. In addition, Puertas and colleagues reported that the protein yield, when using *E. coli* as a host for recombinant production, is inversely proportional to the cysteine content of the protein [113]. This indicates the need for other expression systems when producing proteins with a high cysteine content, such as plant defensins. Yet, production of functional defensins in *E. coli* has been reported. For instance, using *E. coli* both the spruce defensin PgD5 and the Scots pine defensin PsDef1 were produced while displaying a high antifungal activity [114,115]. In line, functional potato snakin-1 (SN1) and defensin-1 (PTH1) were generated using *E. coli* as a host [116].

Nevertheless, peptides produced in eukaryotic systems are often more active, *i.e.*, characterized by a lower MIC-value, as compared to peptides produced in prokaryotic systems. The latter is possibly due to structural defects or misfolding. For instance, Kant and co-workers found that the corn defensin PDC1 exhibited a 2-fold higher antifungal activity when produced in *P. pastoris* as compared to its production in *E. coli*. In addition, Fourier transform infrared spectroscopy (FTIR) revealed more β-sheets and less random structures when PDC1 was produced in *P. pastoris* [117]. Similar observations were made for other proteins. Both human adiponectin and alkaline phosphatase from archaea were found more active when produced in *P. pastoris* as compared to their counterparts in *E. coli* [118,119]. Hence, these observations highlight the advantages of using *P. pastoris* for generation of functional and properly folded proteins.

#### 4.2.2. Heterologous Expression of Proteins in *P. pastoris*

Yeasts are largely used for production of recombinant proteins due to their eukaryotic nature. Unlike bacteria, yeasts have the ability to implement many post-translation modifications, including disulphide bond formation, glycosylation and processing of signal sequences. These features make them attractive hosts for AMP production. Recently, the yeast *P. pastoris* has gained interest as a host for protein production for several reasons, as discussed below [120,121,122,123,124,125,126].

Firstly, *P. pastoris* displays a high growth rate and allows for high cell densities to be reached, resulting in a higher protein yield as compared to yields obtained with other eukaryotic systems (reviewed in [102]). *P. pastoris* is of particular interest for large-scale productions of recombinant proteins, since the growth media are cheap, universal and well defined. Furthermore, when handling fermenter setups in which pH, aeration, feed rate, *etc.* are controlled, *P. pastoris* can easily grow to ultra-high cell densities, which in turn leads to an increased protein yield [127].

Further enhancement of the protein yield can be obtained by using multicopy transformants during protein production (reviewed in [128]). Multiple copies of the plasmid are often incorporated in the *Pichia* genome through crossover events and are integrated in a head-to-tail manner at the same locus. When using proper plasmids such as pPICZ plasmids containing a Zeocin^TM^ resistance gene, multicopy transformants are selected in a straightforward manner by modulating the antibiotic concentration and screening for an increased antibiotic resistance [129].

Thirdly, protein production in *P. pastoris* can be initiated by exogenous addition of inducing agents (reviewed in [126]). Initiation of protein production at any given time point is an asset, since biomass generation, and hence protein yield, is not affected by the potential toxicity of the protein towards the host [127].

Finally, proteins produced by *P. pastoris* are easily exported to the culture medium using signal sequences, such as the *S. cerevisiae* α-factor sequence, which facilitates downstream processing [122,126]. Other signal sequences that direct the protein of interest to the secretory pathway are reviewed by Ahmad and colleagues [126]. A minor drawback associated with the use of the α-factor secretion signal is the presence of protein isoforms in which additional amino acids are incorporated at the N-terminus of the protein due to incomplete processing of the *STE13* protease [126,130]. A non-native N-terminus can influence the biological activity of the protein, as was reported for the pea defensin Psd1 [130,131] and the shrimp AMP Ch-penaeidin [132], and is therefore inadmissible. Addition of an alanine or protease cleavage site at the N-terminus of the protein is recommended as it allows for successful cleavage of the signal sequence [126,130,133,134].

*P. pastoris* has been successfully used for the production of AMPs, including hPAB-β, a variant of human β-defensin, and shrimp Ch-penaeidin [132,135]. Likewise, defensins from pea, tomato, mungbean, Mexican turnip, corn, tobacco, radish, alfalfa and barrel clover were successfully produced in *P. pastoris* [15,40,117,130,133,136,137,138]. These observations highlight the ease of using *P. pastoris* for production of AMPs, and more specifically, plant defensins.

## 5. Conclusions

Plant defensins are interesting candidates for use in medicinal and biotech purposes and can be produced via heterologous expression in eukaryotic hosts. PDFs are generally considered non-toxic to plant and mammalian cells and have distinct modes of action, involving specific interactions with the cell surface [39]. They are therefore suggested to have great therapeutic potential, however, literature falls short on studies reporting the *in vivo* performance of PDFs in an animal model. To our knowledge, only RsAFP2 has been reported to show *in vivo* efficacy in a murine candidiasis model upon intravenous administration [65]. In addition, the mechanisms of action of various PDFs have not yet been identified or are not yet fully understood. Hence, further research is needed to demonstrate the therapeutic potential of PDFs and to elucidate their mechanisms of action.
